# Health care purchasing in Kenya: Experiences of health care providers with capitation and fee‐for‐service provider payment mechanisms

**DOI:** 10.1002/hpm.2707

**Published:** 2018-11-13

**Authors:** Melvin Obadha, Jane Chuma, Jacob Kazungu, Edwine Barasa

**Affiliations:** ^1^ Health Economics Research Unit KEMRI|Wellcome Trust Research Programme Nairobi Kenya; ^2^ World Bank Group Kenya Country Office Nairobi Kenya; ^3^ Nuffield Department of Medicine University of Oxford Oxford UK

**Keywords:** Attributes, capitation, fee‐for‐service, Kenya, provider payment mechanisms

## Abstract

**Background:**

Provider payment mechanisms (PPMs) play a critical role in universal health coverage due to the incentives they create for health care providers to deliver needed services, quality, and efficiency. We set out to explore public, private, and faith‐based providers' experiences with capitation and fee‐for‐service in Kenya and identified attributes of PPMs that providers considered important.

**Methods:**

We conducted a qualitative study in two counties in Kenya. Data were collected using semistructured interviews with 29 management team members in six health providers accredited by the National Hospital Insurance Fund (NHIF).

**Results:**

Capitation and fee‐for‐service payments from the NHIF and private insurers were reported as good revenue sources as they contributed to providers' overall income. The expected fee‐for‐service payment amounts from NHIF and private insurers were predictable while capitation funds from NHIF were not because providers did not have information on the number of enrolees in their capitation pool. Moreover, capitation payment rates were perceived as inadequate. Capitation and fee‐for‐service payments from NHIF and private insurers were disbursed late. Finally, public providers had lost their autonomy to access and utilise capitation and fee‐for‐service payments from the NHIF.

**Conclusion:**

Through their experiences, health care providers revealed characteristics of PPMs that they considered important.

## 1 INTRODUCTION

Sustainable development goal (SDG) 3 places emphasis on health and well‐being of people of all ages and highlights the importance of universal health coverage (UHC) through target 3.8.^1^ UHC means that people, irrespective of their background or financial status, can access quality preventive, promotive, curative, and rehabilitative health services they need and do not suffer any financial consequences, while accessing these services.^2^ Globally, approximately 3.65 billion people lack access to health services that they need.^3^ Furthermore, 800 million incur catastrophic health expenses, while 100 million are pushed into poverty due to health care payments.^3^ The greatest burden is borne by countries in Asia and Africa.^3^

Financing health care is central to achieving UHC as it influences its end goals through three intermediate objectives namely “equity in resource distribution, efficiency, transparency and accountability”.^4^ To reorient health systems towards UHC, LMICs such as Kenya are reforming their health financing strategies. These reforms have largely focussed on revenue collection and pooling functions of health financing. Revenue collection refers to raising funds for health care using various sources such as taxes or insurance contributions while pooling is the accumulation of prepaid funds for health on behalf of a population with the aim of transferring it to providers, to deliver a predetermined set of services.^5^ The eventual transfer of these pooled funds to health care providers to deliver services is known as purchasing.^6^

### 1.1 Health care purchasing

There is growing recognition that purchasing plays a critical role in health financing as it bridges the gap between mobilising revenues for health care, risk pooling, and the eventual delivery of quality services.^7^ Purchasing involves making sets of decisions namely identification of health services to be purchased, selection of health care providers, and determination of how these services will be purchased including contractual arrangements and provider payment mechanisms.^8^, ^9^

A purchaser can include a country's ministry of health, social health insurance scheme, private health insurer, or a different body. Purchasing can either be passive or active.^8^, ^9^, ^10^ In passive purchasing, the purchaser does not continuously use information to allocate the pooled resources.^8^, ^9^, ^10^ For example, allocating funds to a health facility based on the previous year's budget adjusted for inflation or paying bills from providers without checking whether the services were needed.^10^ Active purchasing, also known as strategic purchasing, involves continuously using information to allocate pooled resources such as essential services to be delivered relative to need, the price to pay, quality and quantity of services, and the performance of the staff and facilities.^8^, ^9^, ^10^, ^11^ Countries are moving away from passive and embracing strategic purchasing approaches as these methods have the tendency to improve health systems performance through containing costs, increasing efficiency, and improving access to needed health services.^12^

One of the purchasing decisions involves deciding on the services to be purchased.^8^ This decision entails choosing between less routinely used services such as chronic ailments or routinely used services such as acute illnesses.^5^, ^8^, ^9^ After deciding on the type of services to be purchased, the next decision involves choosing the type of providers that beneficiaries can seek care from.^5^, ^8^, ^9^ Accreditation of health care providers is one of the outcomes of this decision. Finally, in the last purchasing decision, contracts are signed with the selected providers and payment methods are agreed upon.^5^, ^8^, ^9^ These payment methods are known as provider payment mechanisms (PPMs) and are the focus of this paper.

### 1.2 Provider payment mechanisms

PPMs are a key aspect of strategic purchasing as they have the potential of creating incentives for health care providers to improve efficiency, quality, and utilisation of needed health services.^12^, ^13^ The definitions of common PPMs and the incentives they create are outlined in Table 1.

**Table 1:** Definitions of common provider payment mechanisms

Provider Payment Mechanism	Definition	Incentives for Health Care Providers
Salaries	Monthly payments to staff^5^, ^9^, ^14^	No incentive to improve performance, reduce quality, underprovide services^5^, ^15^
Capitation	An advanced fixed payment to a provider to deliver a set of services to an individual (enrolee) for a period of time^9^, ^14^, ^15^, ^16^	Attract more enrolees, select healthier ones, refer patients to other facilities, improve efficiency, control costs, and underprovide services^9^, ^14^, ^15^
Fee‐for‐service (FFS)	A payment for each individual service provided such as consultation, diagnostic tests, or drugs^9^, ^14^, ^15^	Overprovide services above necessary and increase costs^9^, ^14^, ^15^
Global budget	An advanced payment to the facility to cover aggregate expenditures to deliver defined services for a specified period of time^13^, ^14^	Refer patients to other providers, underprovide services, improve efficiency^5^, ^13^, ^14^
Line item budget	An advanced payment to the facility to cover specific line items such as drugs, staff, or supplies^13^, ^14^	Refer patients to other facilities, underprovide services, no incentive to improve efficiency, spend all the money before the end of the financial year^13^, ^14^
Per diem	A fixed payment to provide a set of services to a patient per day^14^, ^15^, ^17^	Increase the number of days a patient is admitted, improve efficiency, reduce quality^13^, ^14^, ^17^
Case‐based payments/diagnosis related group (DRG)	A specific amount paid to provide all services per episode of illness^5^, ^14^, ^17^	Increase admissions more than necessary, discharge patients early^13^, ^14^

### 1.3 Health care purchasing arrangements in Kenya

Kenya has a two‐tier devolved system of governance with a national government and 47 semiautonomous county governments.^18^ The health care system has three purchasing arrangements namely public integrated, public contract, and private contract.^19^ In the public integrated arrangement, the national Ministry of Health purchases health services from tertiary public providers that it owns and pays using global budgets.^19^ The county governments maintain primary and secondary public providers to which they allocate line‐item budgets to deliver services and pay health workers' salaries.^19^ In the public contract model, Kenya's social health insurance, the NHIF, contracts public, private, and faith‐based health care providers, and uses different PPMs to pay for services agreed in the contract for their enrolees.^20^ The NHIF uses capitation to pay for outpatient services and FFS for both outpatient and inpatient services. Furthermore, they use per diem to pay for inpatient services and case‐based payments for benefits that are packaged. Finally, in the private contract method, private health insurers, and community‐based health insurers (CBHI), contract private, public, and faith‐based providers and pay for inpatient and outpatient services using FFS on behalf of their enrolees.^21^

When designing PPMs, the experiences of health care providers with these payment mechanisms are not always put into consideration. For example, an analysis of NHIF's purchasing arrangements in Kenya found that its capitation and FFS payment methods had been poorly designed and implemented as health care providers had neither been involved in setting capitation payment rates nor were their experiences with the reimbursement methods considered.^19^ Furthermore, mechanisms to resolve problems with capitation, such as underprovision of services and FFS's overprovision and high costs to the purchaser, were absent. Therefore, considering providers' experiences is important as it helps in identifying the attributes of PPMs that are important to them. These attributes can sequentially be targets for programmes and interventions that aim to redesign PPMs to create positive incentives for health care providers to deliver needed services, quality, and efficiency.

Evidence of health care providers' experiences with PPMs in sub‐Saharan Africa mostly emanate from Ghana with issues such as delayed reimbursements and inadequate capitation payment rates reported as negative experiences,^22^, ^23^, ^24^, ^25^ and positive experiences such as PPMs adding to the total providers' revenues were also reported.^26^, ^27^ In Kenya, studies focussing on providers' experiences with PPMs are limited. Sieverding et al^27^ explored experiences of private health care providers with NHIF's payment methods and reported that the mechanisms were good revenue sources. However, delayed reimbursements, inadequate capitation rates, poor communication, and support from NHIF negatively affected private providers' participation in the scheme. While the study by Sieverding et al^27^ provides valuable information on PPMs in Kenya, it only focused on private providers and NHIF's payment methods.

To bridge this gap, we set out the experiences of private, faith‐based, and public health care providers with capitation and FFS payment mechanisms in Kenya. We focus on not only providers' experiences with NHIF's capitation and FFS payment mechanisms but also private health insurers' FFS reimbursement schemes. We explore providers' awareness and understanding of the payment methods, their experiences with the reimbursement mechanisms, and their views on how solving the challenges with the payment mechanisms would impact service availability, quality, and efficiency. In the process, we identify the attributes of PPMs that providers consider important and discuss providers' experiences through the lens of these attributes.

## 2 METHODS

### 2.1 Conceptual framework

**Figure 1:**
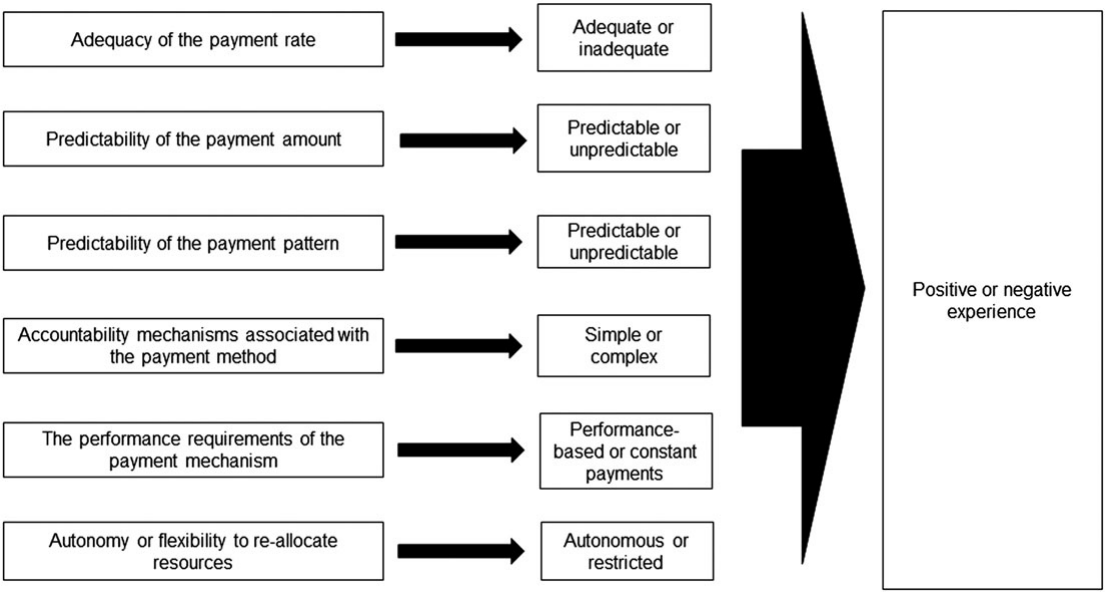
Framework for health care providers' experiences with provider payment mechanisms. Adapted from RESYST consortium's framework on the characteristics of multiple funding flows^28^

With the lack of a framework on experiences of health care providers with PPMs, we adapted the Resilient and Responsive Health Systems (RESYST) consortium's framework on the characteristics of multiple funding flows (Figure 1).^28^ Health care providers' experiences with capitation and FFS payments usually revolve around these characteristics. For example, adequacy signifies how well the payment rate covers the costs of the purchased services. If the payment is adequate, then the provider's experience with the payment mechanism is positive. On the other hand, if the payment is inadequate, then the provider's experience with the reimbursement mechanism is negative.

### 2.2 Study setting

Kenya is a country of 48.46 million people, gross domestic product (GDP) of US$ 70.52 billion, and 36.1% of the population live below the poverty line.^29^, ^30^ Between July 2015 and June 2016, the total health expenditure was US$ 3.48 billion which accounted for 5.2% of GDP.^31^ Within the same period, the government expenditure on health was 6.7% of the total government expenditure, and the government health spending per person was US$ 78.6.^31^

Overall, the country has approximately 10 000 health care providers spread across 47 counties with more than half being public.^32^ About 4000 of these providers are NHIF accredited.^33^ We conducted the study in two Kenyan counties. Table 2 shows the number of health care providers in both counties including those accredited by the NHIF. The NHIF classifies health care providers as public, private, and faith‐based. The two counties reflect existing inequalities in the distribution of health care providers. County A has a high number of public providers compared with private, while county B has the opposite.

**Table 2:** Number of health care providers in County A and B

		Faith‐Based and Nongovernmental Organisations	Private	Public	Total
County A	Providers within the county	18	78	127	223
	NHIF‐accredited providers	11	38	22	71
County B	Providers within the county	62	342	103	507
	NHIF‐accredited providers	21	58	33	112

Source: Authors own analysis of data on health facilities available on the NHIF website^33^ and the Kenya Master Health Facility List.^32^

### 2.3 Study design

We applied a cross‐sectional qualitative approach to explore health care providers' experiences with capitation and FFS payment mechanisms. Qualitative methods allowed in depth examination of providers' experiences.

### 2.4 Sampling

We purposely selected two counties to reflect existing inequalities in the distribution of health care providers. Next, we purposely selected six health care providers (two private, two public, and two faith‐based) from the list of NHIF‐accredited providers that offered both inpatient and outpatient services. We selected NHIF‐accredited providers due to their existing exposure to multiple PPMs. Moreover, NHIF was the main health insurance provider covering 80.65% of all insured Kenyans who are often coinsured with private or community‐based health insurance.^34^ We defined a health care provider as a facility or hospital that provided health care services.

Selected health care providers were contacted through emails, phone calls, and face‐to‐face visits, and consent to participate in the study sought from institutional heads. Within each of the six health care providers, we purposely selected five members of the management team who made financial and budgetary decisions on PPMs to participate in the face‐to‐face interviews. Members of the senior management team were selected in private and faith‐based health care providers and health management team (HMT) members in public providers. The total sample size was 30. One senior manager at a private provider declined to participate citing a busy schedule even after two repeated attempts. Table 3 shows the characteristics of health care providers who participated in the study.

**Table 3:** Characteristics of the health care providers and interview respondents

County	Health Care Provider Type	Interview Respondent	No.
County A	Public Secondary level	Medical superintendent	1
		Pharmacist‐in‐charge	1
		Hospital administrative officer/accountant	1
		Nursing officer‐in‐charge	1
		Clinical officer‐in‐charge	1
	Faith‐based Secondary level	Medical director	1
		Administrative director	1
		Matron‐in‐charge	1
		Finance manager	1
		Pharmacist in‐charge	1
	Private Primary level	Medical director	1
		Administrative director	1
		Matron in‐charge	1
		Pharmacist in‐charge	1
		Total	14
County B	Public Secondary level	Medical superintendent	1
		Pharmacist‐in‐charge	1
		Hospital administrative officer	1
		Nursing officer‐in‐charge	1
		Hospital accountant	1
	Faith‐based Secondary level	Medical director	1
		Administrative director	1
		Matron‐in‐charge	1
		Finance manager	1
		Pharmacist in‐charge	1
	Private Secondary level	Medical director	1
		Administrative director	1
		Matron in‐charge	1
		Pharmacist in‐charge	1
		Hospital accountant	1
		Total	15
		Grand total	29

### 2.5 Data collection

Data were collected using semistructured interviews between late September 2017 and early December 2017. The interview guide was developed using the study framework (Figure 1). The guide covered areas such as awareness and understanding of provider payment mechanisms, positive and negative experiences, characteristics of capitation and FFS payments, and implications of redesigning payment methods on service availability, quality, and efficiency. The guide was piloted among different providers in county A and revised in an iterative process.

We conducted face‐to‐face semistructured interviews with 29 respondents at their place of work after obtaining written informed consent from them. All interviews were conducted in English, audiorecorded, and lasted between 30 and 50 minutes. We additionally wrote field notes during and after the interviews. Two researchers transcribed all the interviews verbatim and checked for errors and inconsistencies. Data saturation was achieved.

### 2.6 Data analysis

We used a framework approach to analyse transcribed data. Two researchers familiarised themselves with the transcripts by repeatedly reading them. A framework was developed from the study objectives and conceptual framework (Figure 1) with emerging themes being included from the transcripts. One researcher developed the coding scheme derived from the data and framework which was reviewed and revised by two other researchers. We used NVIVO 10 to code and chat transcripts. Finally, three analysts then mapped and interpreted the findings.

### 2.7 Ethical approval

The study received ethical approval from the Kenya Medical Research Institute/Scientific and Ethics Review Unit (KEMRI/SERU) under SSC No. 2795.

## 3 RESULTS

### 3.1 Health care providers had a good understanding of capitation and FFS payment mechanisms

Health care providers' understanding of capitation and FFS was gauged on whether they could identify the following four aspects of a payment mechanism namely the purchaser, on whose behalf the payments were made, services the PPM was paying for, and rate and frequency of payment.

Providers had a good understanding of capitation and FFS payment mechanisms and how they worked. They identified two purchasers namely NHIF and private health insurers. Respondents noted that the NHIF used capitation to pay for outpatient services and FFS to pay for inpatient care. Additionally, providers mentioned that private insurers used FFS to pay for both inpatient and outpatient care.

Respondents indicated that the NHIF capitation amounts varied according to the ownership of health care providers (private health care providers received a higher rate compared with public providers) and scheme (the NHIF civil servants scheme had a higher capitation rate per enrolee than the general scheme). Moreover, respondents reported that the NHIF disbursed capitation payments to health care providers quarterly, while FFS reimbursements were paid after submitting claims to either the NHIF or private health insurers in accordance to the contracts signed between the providers and purchasers. As for FFS payments, providers stated that the rates paid were equivalent to the services offered as indicated in the claims, and payment periods differed according to the contracts they had signed with purchasers.

“When you are an NHIF client and have chosen [this] hospital, what happens for outpatient services, we are being given some capitation … If you are a single individual, they are paying I think 100 shillings [US$ 1] per month … So, the whole year, they are paying 1,200 [US$ 12] per individual. So, if you are a family of three, we will calculate 300 [US$ 3] times 12 months... And they are paying us quarterly … You just subdivide that from January to April. So there, they pay us 400 [US$ 4]. And then you count April to August, they pay 400 [US$ 4]. Then from that to December they [pay] like that.” – Senior manager 5|faith‐based provider ( County A).

“When the patients come in, they are given all the services that they need. And then, we will fill an invoice. We invoice the [private] insurance company. We send them the bill monthly” – Senior manager 2|private provider ( County B).

### 3.2 Capitation and FFS payments were considered a significant contributor to overall providers' revenues

The extent to which PPMs contributed to the overall revenue envelope was important to health care providers. Capitation payments from the NHIF and FFS payments from both the NHIF and private insurers were generally viewed as good sources of revenue by public, private, and faith‐based providers. Capitation and FFS payments from NHIF were considered good because they were guaranteed despite delays and came in lump sum thereby enabling them to finance their budgets.

“Let me say, the positive [is] that this money [NHIF capitation and FFS] comes in a lump sum. Because they come either at the start of the month or at the end of the month. The moment it comes in a lump sum, we have got big revenue for our budget and services”. ‐ HMT member 2|public provider ( County B)

Furthermore, FFS payments by private insurers were also deemed to be good sources of income as they sustained faith‐based and private providers and contributed to their total revenues.

“The positive aspect is that we have a good relationship with the corporate [private] insurance … We get many patients... That is what sustains us”. – Senior manager 2|faith‐based provider ( County B).

### 3.3 The capitation payments by NHIF were considered inadequate

The adequacy of the PPM to cover the costs of services provided was also a concern to health care providers. NHIF capitation rates per individual were viewed as inadequate and did not reflect the cost of services provided by health care providers. Specifically, providers mentioned that the rates per enrolee were inadequate for chronic conditions such as hypertension and diabetes.

“But the capitation itself which is given in most of the facilities across the board including this facility that we are talking about is low. It is inadequate … I would say for example [this] facility if it is 1,200 [US$ 12] per family, one of the family members can come and the outpatient services would consume that with only one test”. – Senior manager 4|faith‐based provider ( County A)

Public health care providers complained of receiving lower capitation rates per enrolee as compared with private and faith‐based providers and that the rates were not set in consultation with them.

“That is where the problem is because when they [NHIF] are coming with a cost per person, they are supposed to bring a stakeholder on board so that we agree. They just decide for themselves and this is where they favour, especially the NHIF, they favour private hospitals at the expense of the government hospitals. Whereas we are offering same services” – HMT member 2|public provider ( County B)

Some providers suggested that purchasers like NHIF should consider increasing capitation rates per individual as it would motivate them to provide quality‐needed services.

“If the money that we are given is sufficient, we will be able to purchase the needed medicine … Hence, we will be able to offer better services. The quality service to the patient”. – Senior manager 1|faith‐based provider ( County B)

### 3.4 Capitation payments from the NHIF and FFS payments from the NHIF and private insurers were delayed

Another attribute of importance to health care providers was the predictability in timing of payment disbursements. Delays in receiving capitation and FFS reimbursements from both the NHIF and private health insurers were strongly reported by all health care providers. These delays affected providers' operations such as salary payments, purchase of commodities, and implementation of work plans. Furthermore, there was poor communication from the purchasers concerning delays in reimbursements.

“Private sector [private health insurers] their payment is very poor and very late... some of the cases even go for a whole year … That is private insurance across the board. You have to keep on following up all that time”. – Senior manager 1|private facility ( County B)

“I kid you not. Sometimes we get the payments [NHIF capitation and FFS] even a year later from when we expected to get these payments. … That renders us indebted to our suppliers.”. – HMT member 4|public provider ( County A)

Private, public, and faith‐based providers wanted a PPM that reimbursed funds on time for both capitation and FFS payments. Knowing when they would be paid would assist them in planning, purchasing equipment, and commodities, and providing services.

“If I get this money from the from the [NHIF] insurance in time, I will make all the reagents in the lab available. I will make all the essential drugs available. And if I have got a staff shortage, I can as well use that money maybe to employ on contract bases like clinical officers, laboratory, and even nurse” – HMT member 1|public provider ( County A)

### 3.5 The expected amount of funds disbursed to health care providers was more predictable for FFS from both the NHIF and private insurers but unpredictable for capitation payments from the NHIF

Health care providers were sensitive to the predictability of amounts disbursed. Predictability in the amount means that providers know how much to expect. Public, private, and faith‐based providers had good experiences with both FFS payments from NHIF and private health insurers as they could easily predict the amount to expect and had no problems with reimbursement rates. What made the expected FFS payment amounts predictable according to providers was the fact that the funds paid by purchasers were equivalent to the services offered by providers as indicated in the claim forms. This aspect of FFS' predictability was advantageous to providers as it ensured that they could easily budget and plan for their activities.

“… fee‐for‐service for the private [health insurance], we do not have an issue with it because they usually tell us how much they are paying and they will pay exactly that amount... So, the private company we usually do not have issues with them. The NHIF, the fee‐for‐service is only for the civil servant and they pay everything. So, we do not have an issue with that”. – Senior manager 1|faith‐based provider ( County B)

However, health care providers could not predict the expected total capitation payments from the NHIF because they did not know the number of enrolees they had in their capitation pool. This was because the list of enrolees registered to their health care facilities was not availed to them by NHIF. Providers could query online on the NHIF information system whether an individual was registered to their facility but could not view the whole list of enrolees. This raised the suspicion that they were serving more enrolees than the total amounts paid to them.

So maybe, if there could be a system for us to know that is the list [list of the people registered to their health facilities]. That these are the number of clients who are supposed to be treated at our facility, it would be better. So that we also know how they arrive at this [capitation] figure. Maybe it starts with NHIF. They can tailor make their system such that we are given an access to wherever the registration has taken place so that we are able to know who is supposed to be treated at our facility... But we do not know the total number so that we know the figure of capitation per quarter – Senior manager 1|faith‐based provider ( County A).

Furthermore, public, private, and faith‐based providers thought that the NHIF tampered with the list of enrolees. Public providers also thought that the NHIF colluded with private providers to tamper with the list and reallocate individuals from public to private providers.

“There are people who choose this hospital. Then, they [NHIF] go and change and give them to private hospitals. Do you know why? Because private hospitals they can easily risk their hands and you know government hospitals there are a lot of bureaucracies. So, there is that complain. But when I went to the [NHIF] office, there were those young guys who were employed there. I think they are the people who are doing that. They were colluding with those private hospitals. So, that is why we always want a list of everybody.” – HMT member 1|public provider ( County A)

“Somebody comes and he is not on your list of clients and I'll tell him you are not on the list of clients. Then, he goes back [to NHIF offices] and comes back after a few hours and says I am told that I can now get this service from here. And then again if you check, you find the name now has appeared. I think a bit of dishonesty... because I think it is possible for them [NHIF] to do that.” – Senior manager 3|private provider ( County A)

Providers desired to have access to the full list of enrolees registered to their facilities under NHIF capitation. This would help them to predict the total capitation amounts expected, plan, and provide needed services.

“If they [NHIF] could reveal to us the numbers that have actually chosen our facility, it will also help us in our budgetary work. So that I know this quarter, I will be catering for outpatient of about 1000 people. And out of that 1000 people, maybe 500 may not come. I will be able to purchase [drugs] because I know what ails people on a daily basis... Because in this area, basically, it is malaria, diarrhoea, you know these ailments. And these drugs you can buy them from the wholesale and you buy them at a cheaper price” – Senior manager 1|private provider ( County A)

### 3.6 Public providers had lost their autonomy to access and utilise funds from capitation and FFS payments from the NHIF

The autonomy to access and utilise PPM funds was of great significance to health care providers. Private and faith‐based providers had the autonomy to access and use FFS and capitation funds from NHIF and private insurers while public providers had lost this autonomy. This is because private and faith‐based providers received these payments directly into their own accounts. It meant that they could access and use the money at any time to pay salaries and purchase commodities such as drugs.

“It is good that once money is given, then the facility can see how best they can maximise the use of that fund depending on the kind of ailments the clients have” – Senior manager 4|faith‐based provider ( County A)

Public providers, on the other hand, lacked the autonomy due to the restrictions imposed on them by the Kenyan Public Finance Management Act of 2012. Once they received capitation and FFS payments from the NHIF, they first had to deposit the funds into the county revenue fund and then claim it back. It would take months for the county to refund the cash, and they would in most cases not refund the full amount. Since disbursement of the NHIF funds would most probably have been delayed, refunds from the county would cause further delays. This affected public providers operations such as payment of suppliers, purchase of drugs, and commodities.

“According to the hospital regulations, we are not supposed to use that money until we transfer it to the county revenue account in order for them again to reimburse us. Can you see there is that gap?... We cannot use that money. So, again when it goes to the county government, they cut the 5%... Then they give us when it is not 100%.” – HMT member 3|public provider ( County B)

Public health care providers mentioned that receiving funds directly into their accounts and not having to deposit it into the county revenue fund would hasten the process of paying suppliers and settling debts. This would improve their capacity to provide additional services through financing their own facility improvement projects.

“We think the best way is... for the county government to let us bank in our own hospital accounts. That one will at least help us to reduce delay in the payment of suppliers. We will reduce our debts as a hospital and many other things. We would have even major, major projects going on.” – HMT member 3|public provider ( County B)

### 3.7 Complex NHIF reporting requirements led to monetary losses

The complexity of reporting requirements was a concern to health care providers. Reporting requirements for FFS payment claims by NHIF were viewed as complex by private, public, and faith‐based health care providers. NHIF required providers to fill claim forms, upload them onto the online system, and present paper copies to their offices for verification and approval. If the details on the online system did not tally with those on the paper copy, the payments got rejected and providers would incur monetary losses. The double reporting was termed unnecessary by the providers.

“Again, the forms which we fill. You see, they [NHIF] provide us with one form... Then you go and register [file the claim] online. Again, you make a phone call and then you take it [paper copy] there. And then they verify it there to approve it. “Tell me, what is the of point having an online system? Just do it on the paper and give it … Why do you have to do it again? You do it here and they do it there. And when they [NHIF] misplace one, my claim is rejected … There is a claim which we gave in time. There was some problem with it. It was not verified. 72,000 shillings [US$ 720]. I lost it because they are saying we did not put it in time. The system does not take it now. I'm to be blamed, I am losing … We are in digital time and yet we have difficulties and it is a problem.” – Senior manager 3|private provider ( County B)

Furthermore, providers had to notify the NHIF through the online system that an enrolee had sought care from their facility. Providers had to do this within a short time frame from when the NHIF enrolee sought care or they would risk not being reimbursed the FFS claims. This also meant that in times of system outages, providers did not have alternative means of notifying NHIF. Therefore, if they provided any services during that period and were not able to log the details on the system in time, then NHIF would not reimburse them.

“Again, there are these payments that you need to notify using the system. If the network is poor either from our end or their end, you suffer here at the site. If their system is not working, we are affected. So, you will come with an active NHIF card, we cannot see you because their system is low. That means we cannot offer you the service. But you are eligible for that service. You have an active card that needs to receive that service. But now, if their system is not working or our system is not working, we cannot work. If we give the service and we realise from their end it is not active, then we run the loss. So those kinds of infrastructural problems they affect service to the client who probably needed that service.” – Senior manager 3|faith‐based provider ( County A)

Private, public, and faith‐based providers desired simple, accurate, and user‐friendly reporting requirements and alternative methods of notifying NHIF and sending FFS claims during system downtimes. This would reduce bureaucracies and increase efficiency.

“It needs to be simple in the context that the processes of right from when a client subscribes, to when the service is offered in the hospital, to when the hospital demands payment, to when the provider [NHIF] eventually releases payment to the hospital. That needs to be simplified. Minimal bureaucracies.” ‐ HMT member 4|public provider ( County A).

### 3.8 FFS payments from private insurers were problematic when tied to performance indicators

Performance requirements were also another key attribute of a PPM according to health care providers. FFS payments from private insurers were viewed as problematic by private and faith‐based providers when tied to performance indicators such as quality. One of the quality indicators imposed by private insurers was complications arising from procedures such as surgeries. Private insurers would not pay for such cases especially when the costs escalated.

“For the fee‐for‐service, the main disadvantage is probably if there are complications arising and the hospital incurs higher cost and the insurer feels those costs were not part of the amount that they had covered … Sometimes the insurance may be hesitant in covering those high bills.” – Senior manager 3|faith‐based provider ( County B)

Faith‐based and private providers suggested if private insurers would be flexible in dealing with quality performance‐based payment issues such as complications arising from care.

“For example, in the long stay patients, one of the things is for the insurance to be flexible and to communicate with the hospitals. They should always make sure that they clearly tell the hospitals to inform them early enough if there are complications arising from inpatient care. For example, with surgeries is very common. Patients who go in for repeat surgeries for example, they should be able to alert the hospitals in good time and the hospital also has obligations to send information immediately there is a complication.” – Senior manager 3|faith‐based provider ( County B)

## 4 DISCUSSION

We set out to explore the experiences of health care providers with capitation and FFS payment mechanisms in Kenya. We focused on providers' awareness and understanding of the payment methods, their experiences with the mechanisms, and their views on how solving the challenges with the payment mechanisms would impact service availability, quality, and efficiency. In the process, we identified the attributes of PPMs that providers considered important and discuss providers' experiences through the lens of these attributes.

Health care providers generally understood how capitation and FFS payment systems worked. Our results differ from other studies that found that health care providers in Kenya had a low level of awareness about provider payment mechanisms.^27^, ^35^ This difference is likely because our study interviewed senior managers who were responsible for financial planning and decision making. Other studies interviewed frontline health care workers. Interestingly, in spite providers in our study exhibiting high awareness levels of PPMs, there were some instances where they did manifest lack of knowledge. For example, providers did mention that the NHIF capitation rates per enrolee differed by the provider type with private providers receiving a higher rate per enrolee as compared with faith‐based and public. According to the NHIF, capitation rates per enrolee paid to providers were standardised in July 2017 and meant that public, private, and faith‐based providers were all paid a single rate of KES 1200 (US $12) per enrolee for the general scheme and KES 2850 (US $28.50) for the civil servants' scheme. This observation might imply that providers did not fully understand how PPMs worked or the NHIF did not provide full information to them on how the scheme works.

Through their experiences, we found that health care providers considered several attributes of PPMs as being important. The extent to which PPMs contributed to the overall revenue envelope of the providers was important. Capitation and FFS payments from NHIF and private insurers were considered good sources of revenue for the providers as they significantly contributed to their overall revenues when received in a lump sum. Similar findings were reported in Ghana and Kenya. In Kenya, NHIF's payments including capitation and FFS added to providers' income and increased patient flow to the facility.^27^ In Ghana, National Health Insurance Scheme (NHIS) payments received by bulk assisted providers to undertake major infrastructural projects and avail services.^22^

Public, faith‐based, and private providers were also sensitive to the predictability of the timing of payment disbursements. Delays in payment negatively affected providers performance as they could not plan, pay worker salaries on time, and purchase commodities such as drugs and equipment. Delayed reimbursement of FFS funds from private insurers and NHIF and capitation payments from NHIF created incentives for providers to discriminate and ration services to the insured which affected the availability of needed services. Similar observations were reported in Nigeria where unpredictable payment patterns from the free maternal and child health care programme led to drug stockouts in public facilities which prompted providers to introduce informal payments for services that were otherwise free.^36^ In India, delayed FFS reimbursements from the National Health Insurance Scheme (Rashtriya Swasthya Bima Yojana) forced health care providers to turn away enrolees and deregister themselves from the scheme.^37^

Health care providers were also concerned about the predictability of amounts disbursed by PPMs. Public, private, and faith‐based providers complained that expected amounts under the NHIF capitation were unpredictable because they did not have knowledge of the number of enrolees registered to their facilities. This scenario was also reported in Nigeria by Mohammed et al^38^ where 30% of health care providers in their study did not receive the list of enrolees capitated to their facilities from the National Health Insurance Scheme on a quarterly basis as expected. The lack of transparency in revealing the list of enrolees is clearly important as it is linked to the predictability of capitation payment amounts. The fact that the NHIF did not share the full list of enrolees capitated to health care facilities with providers as revealed by our study, bred mistrust between them and the providers and between public and private providers. In Kenya, Abuya et al^39^ noted the perceived lack of transparency in NHIF operations especially by private sector actors contributed to distrust of the organisation.

The adequacy of PPM rates was another important aspect. Though public, private, and faith‐based providers in our study liked FFS payment rates from private health insurers and NHIF because they could make profits, the reimbursement method is costly and inefficient to the purchaser and health system. In Thailand for example, FFS payments from the Civil Servant Medical Benefit Scheme created incentives for providers to over provide nonessential drugs between 2011 and 2012, leading to high reimbursement rates, with a single health facility receiving an average reimbursement amount of $ 495 per patient for drugs alone.^40^ Our study also revealed that public, private, and faith‐based health care providers were dissatisfied with NHIF capitation rates because they deemed them to be inadequate. This resonates with findings from other settings such as Burkina Faso where Robyn et al^41^ found that capitation rates paid by a CBHI scheme were inadequate to cover the cost of services provided to enrolees. Interestingly, providers in our study mentioned the fact that capitation rates were inadequate especially for chronic conditions such as diabetes and hypertension. What providers did not know was that they were supposed to claim separately for these chronic conditions. This might be due to limited information provided by the NHIF.

Health care providers were also sensitive to the complexity and burden of reporting and claims mechanisms of PPMs. The complex reporting requirements for the NHIF FFS payments were considered burdensome to health care providers. Similar experiences were observed in Karnataka, India, where Rajasekhar et al^42^ found that the National Health Insurance Scheme (Rashtriya Swasthya Bima Yojana) imposed complex reporting requirements on health care providers such as the use of smart card technologies and computerised reimbursement systems and did not provide adequate training on how to use the system or support. System problems contributed to delays in receiving FFS reimbursement which forced providers to deny patient treatment, implement balance billing and copayments which the patient claims later, or withdraw from the scheme altogether.

Kenya's experience shows that the autonomy that health care providers have over resources is important. Devolution arrangements and a new public finance management law resulted in the reduction of autonomy that public health care providers had. These sentiments are reinforced by Barasa et al^43^ who found that public providers lost the autonomy over finances with the onset of devolution in Kenya. This demotivated public health care providers which in turn compromised quality of health services. In Tanzania, financial autonomy was provided to public providers through allowing them to receive payment‐for‐performance funds directly into their accounts rather than the district managers' accounts. As a result, transparency improved which in turn motivated public providers to improve quality and efficiency.^44^

Finally, we found that if providers' preferences were put into consideration when designing PPMs, it would motivate them to avail quality‐needed services, improve quality, and efficiency. Predictable payment amounts, timely payment patterns, adequate capitation rates, autonomy, and simple reporting mechanisms can modify providers' behaviours and create positive incentives.

Drawing from our findings, we make several recommendations on how to improve strategic purchasing for health care in Kenya through strengthening PPMs. First, in response to the perceptions of the inadequacy of the NHIF capitation rate, the NHIF should embrace best practice in the setting of PPM rates. Specifically, the capitation rate should be developed based on evidence from costing studies and engagement with public, private, and faith‐based health care providers. This is because while the concerns about the inadequacy of the capitation rate may be legitimate, they may be caused by a lack of understanding of the mechanics of the capitation payment system. According to literature from LMIC settings, providers often complain of the inadequacy of capitation payments because they understand the rate to be an amount that should cover the cost of an individual patient rather than a risk‐adjusted amount aimed at covering the health care costs of a pool of individuals whether or not they seek care.^24^, ^25^, ^27^, ^38^, ^41^ Second, both the NHIF and private health insurers should improve the timeliness of their fund's disbursement to health care providers. This will improve the planning and operations of health care providers. Third, the NHIF should improve the transparency of the operations of the capitation payment mechanism. Specifically, the list of enrolees registered to a health care facility should be shared with providers so as to improve the predictability of the expected amounts. Fourth, the government of Kenya should consider amending the public finance management act to allow public health care providers to operate bank accounts and have the autonomy to spend funds received from the NHIF. Alternatively, county governments can pass legislation that allows public providers within those counties to retain the funds. Lastly, the NHIF should simplify its claims and reporting processes to improve health care provider experiences and lessen their burden on accountability, reduce duplication in activities, and thus improve efficiency.

The study highlights aspects to consider while designing PPMs, albeit with some limitations. Since the sample only included NHIF‐accredited providers, we missed out on capturing nonNHIF‐accredited providers' experiences with FFS payments from private health insurers. In addition, our study is only context specific and might not be generalisable to other settings. However, our results are useful in informing design of PPMs.

## 5 CONCLUSION

PPMs are a potential power lever in health system reform since their configuration influence the behaviour of public, private, and faith‐based health care providers. This study has not only illuminated the experiences of health care providers in Kenya with PPMs but, through doing so, has identified a range of attributes of PPMs that health care providers consider important. These attributes are therefore potential targets for interventions aimed at configuring PPMs to achieve health system goals. While we have identified these attributes as important, this study does not reveal their relative importance which is useful in informing trade‐offs in intervention design. Further research to elicit the relative importance of these attributes, such as through discrete choice experiments, could answer this question.

## DECLARATIONS

## ETHICS APPROVAL AND CONSENT TO PARTICIPATE

The study received ethical approval from the Kenya Medical Research Institute/Scientific and Ethics Review Unit (KEMRI/SERU) under SSC No. 2795. Furthermore, the National Commission for Science, Technology and Innovation (NACOSTI) gave clearance for the study to be conducted. Finally, all the participants signed the informed consent form before being interviewed.

## CONSENT FOR PUBLICATION

Consent to publish findings of the study was obtained from the participants of the study.

## AVAILABILITY OF DATA AND MATERIALS

The data generated and analysed during the current study are not publicly available due to them containing information that could compromise research participant privacy. However, the transcripts are available from the corresponding author [MO] on reasonable request.

## AUTHOR CONTRIBUTION

EB and JC conceptualised the study. The interview guide was developed by MO, JK, and EB. Data were collected by MO and JK. MO developed the coding tree which was reviewed by EB and JK. Coding, charting, and mapping were conducted by MO with EB and JK contributing in the interpretation of findings. The initial manuscript was drafted by MO which was subsequently revised in collaboration with EB, JK, and JC. All authors read and approved the final manuscript.

## CONFLICT OF INTEREST

The authors declare no conflict of interest.

## FUNDING

MO and JK are supported by a Wellcome Trust grant intermediate fellowship awarded to JC (#101082). EB is supported by a Wellcome Trust training fellowship (#107527). Funds from the Wellcome Trust core grant (#092654) awarded to KEMRI‐Wellcome Trust Research Program also supported this work. The funders and the World Bank had no role in the study design, data analysis, decision to publish, drafting, or submission of the manuscript.
